# The reef-building coral *Siderastrea siderea* exhibits parabolic responses to ocean acidification and warming

**DOI:** 10.1098/rspb.2014.1856

**Published:** 2014-12-22

**Authors:** Karl D. Castillo, Justin B. Ries, John F. Bruno, Isaac T. Westfield

**Affiliations:** 1 Department of Marine Sciences, University of North Carolina at Chapel Hill, Chapel Hill, NC 27599–3300, USA; 2 Department of Marine and Environmental Sciences, Marine Science Center, Northeastern University, Nahant, MA 01908, USA; 3 Department of Biology, University of North Carolina at Chapel Hill, Chapel Hill, NC 29599–3280, USA

**Keywords:** tropical scleractinian coral, calcification, ocean warming, ocean acidification, *Siderastrea siderea*, Caribbean

## Abstract

Anthropogenic increases in atmospheric CO_2_ over this century are predicted to cause global average surface ocean pH to decline by 0.1–0.3 pH units and sea surface temperature to increase by 1–4°C. We conducted controlled laboratory experiments to investigate the impacts of CO_2_-induced ocean acidification (*p*CO_2_ = 324, 477, 604, 2553 µatm) and warming (25, 28, 32°C) on the calcification rate of the zooxanthellate scleractinian coral *Siderastrea siderea*, a widespread, abundant and keystone reef-builder in the Caribbean Sea. We show that both acidification and warming cause a parabolic response in the calcification rate within this coral species. Moderate increases in *p*CO_2_ and warming, relative to near-present-day values, enhanced coral calcification, with calcification rates declining under the highest *p*CO_2_ and thermal conditions. Equivalent responses to acidification and warming were exhibited by colonies across reef zones and the parabolic nature of the corals' response to these stressors was evident across all three of the experiment's 30-day observational intervals. Furthermore, the warming projected by the Intergovernmental Panel on Climate Change for the end of the twenty-first century caused a fivefold decrease in the rate of coral calcification, while the acidification projected for the same interval had no statistically significant impact on the calcification rate—suggesting that ocean warming poses a more immediate threat than acidification for this important coral species.

## 1. Introduction

Atmospheric *p*CO_2_ has increased from pre-industrial levels of *ca* 280 µatm to current levels exceeding 400 µatm [1,2], primarily due to the burning of fossil fuels, cement production and deforestation. This anthropogenic elevation of atmospheric *p*CO_2_ has already decreased surface ocean pH by *ca* 0.1 pH unit [3]. Atmospheric *p*CO_2_ is predicted to exceed 600 µatm by the end of the twenty-first century [4], which would cause surface ocean pH to decline by an additional 0.3 pH units [5,6]. This process of ‘ocean acidification’ reduces the carbonate ion concentration of seawater, which in turn reduces its saturation with respect to the calcium carbonate mineral aragonite, from which scleractinian corals and other marine invertebrates and algae build their protective shells and skeletons.

Atmospheric *p*CO_2_ is also a greenhouse gas and its elevation has caused sea surface temperatures within the habitats of tropical scleractinian corals to increase by as much as 0.7°C over the past several decades [7,8]. The relationship between seawater temperature and calcification rates of tropical corals has been well explored [9–12]. In general, calcification rate increases with increasing seawater temperature up to an optimal temperature, which typically coincides with the mean summer seawater temperature of the coral's natural habitat [11]. At sufficiently elevated temperatures, corals lose their symbionts through a process known as bleaching, resulting in a further decline in calcification. Because maximum summertime temperatures on tropical reefs already approach the temperature at which corals bleach [13], even a small increase in average seawater temperature may negatively impact their fitness.

The number of studies investigating the impacts of ocean acidification on coral calcification has increased exponentially [14–20], with several reviews published on the subject [21–23]. With each additional study, it is increasingly apparent that the calcification response of scleractinian corals to ocean acidification varies widely among taxa [16,20,24,25], and can vary within the same coral species when other experimental parameters (e.g. feeding, light, temperature, method of acidification) are modified [20,24]. Many of these experimental studies have shown that calcification rates of scleractinian corals decline relatively linearly with reductions in seawater pH [15,16,19,26–35]. However, other experimental studies have shown that scleractinian corals can also exhibit no response, a nonlinear threshold response or even a positive response to CO_2_-induced reductions in seawater pH [14,18,36–39]. The complexities of the relationship between seawater pH and calcification rates of scleractinian corals are compounded by interactions between thermal and pH stress that are still not fully understood. For example, the negative effects of reduced seawater pH on coral calcification have been shown to increase under elevated temperatures, suggesting a synergistic effect [30,36,40], while other studies have shown that elevated temperature has either no effect or a mitigating effect on the response of scleractinian corals to ocean acidification [25,29,38,41,42]. This variability in corals’ calcification response to ocean acidification, compounded by the interactive effects of other stressors, complicates efforts to predict and potentially mitigate the impacts of CO_2_-induced ocean acidification on coral reefs.

Although the most adverse impacts on corals may arise from the combined effects of acidification and warming, the objective of this study was to isolate the impacts of these two stressors. Here, we present results of 95-day laboratory experiments designed to investigate the impacts of CO_2_-induced ocean acidification (*p*CO_2_ (s.d.); 324 (89), 477 (83), 604 (107) and 2553 (506) µatm) and warming (temperature (s.d.); 25 (0.14), 28 (0.24) and 32 (0.17)°C) on calcification rates of the tropical reef-building zooxanthellate coral *Siderastrea siderea*—an important and ubiquitous component of Caribbean reef systems [43].

## 2. Material and methods

### (a) Coral collection, transportation and maintenance

In July 2011, eighteen 20–30-year-old colonies of *S. siderea* were collected by hammer and chisel at 3–5 m depth from near shore, backreef and forereef reef zones in southern Belize [8] (see the electronic supplementary material for a detailed description of coral collection sites). Whole corals were transported to the Aquarium Research Center at the University of North Carolina at Chapel Hill by aeroplane. At UNC-Chapel Hill, each coral colony was sectioned into 18 comparatively sized specimens (surface area: 3 × 2 cm; thickness: 1 cm) with a diamond-embedded petrographic saw and glued with cyanoacrylate to acrylic microscope slides. The coral specimens were allowed to recover for 30 days under laboratory conditions in two 500 l recirculating artificial seawater systems maintained at a salinity of 35, temperature of 28°C and an irradiance of *ca* 250 µmol photons m^−2^ s^−1^. The corals were visually inspected each day of the recovery period and no evidence of bleaching or disease was observed. The corals were then acclimated for 15 days following the recovery period, after which the coral specimens were incrementally exposed to the modified *p*CO_2_ and thermal conditions.

### (b) Growth conditions

#### (i) Ocean acidification experiment

*Siderastrea siderea* coral specimens from each of the 18 colonies were reared for 95 days (5 August–8 November 2011) in each of twelve 38 l glass tanks (18 specimens per tank; 216 specimens in total) filled with artificial seawater formulated at a salinity (s.d.) of 35.13 (0.32) with *Instant Ocean Sea Salt* and deionized water. Although the trace elemental composition of *Instant Ocean Sea Salt* differs subtly from that of natural seawater, its major and minor elemental composition and its carbonate chemistry are the most similar to natural seawater when compared with eight other commercial sea salt mixes [44]. Four CO_2_ partial pressures (s.d.) (324 (89), 477 (83), 604 (107), 2553 (506) µatm)), corresponding to a near-pre-industrial, a near-present-day, an end-of-century and an extreme year 2500 *p*CO_2_ level were selected to define the shape of the *p*CO_2_-calcification response curve for *S. siderea*. CO_2_ partial pressures were established by mixing pure CO_2_ with CO_2_-free compressed air (CO_2_ was removed with a Parker Hannifan FTIR Purge Gas Generator) using high-precision digital solenoid-valve-based mass flow controllers (Aalborg Instruments and Controls; Orangeburg, NY, USA). The experimental seawater was bubbled with microporous ceramic airstones into triplicate glass tanks (12 total). The *p*CO_2_ of the mixed gases was measured with a Qubit S151 infrared *p*CO_2_ analyser (Qubit Systems; Kingston, Ontario, Canada) calibrated with certified air-CO_2_ gas standards (precision = ±2.0%; accuracy = ±1.8%). Coral specimens from the 18 colonies were reared in each of the 12 replicate tanks. The *p*CO_2_ treatments were maintained at an average temperature (s.d.) of 28.10 (0.28)°C.

#### (ii) Temperature experiment

Experimental growth conditions for the temperature experiment were similar to those for the acidification experiment described above. *Siderastrea siderea* coral specimens from each of the 18 colonies were reared for 95 days (5 August–8 November 2011) in each of nine 38 l glass tanks (18 specimens per tank; 162 specimens in total) maintained at seawater temperatures (s.d.) of 25.01 (0.14), 28.16 (0.24), and 32.01 (0.17)°C. Salinity (s.d.) was maintained at 35.01 (0.12) by dissolving *Instant Ocean Sea Salt* in deionized water. These temperatures correspond to the corals' approximate annual minimum, mean and maximum seawater temperature as determined from more than 10 years (2002–2014) of *in situ* seawater temperature records obtained near the coral collection sites [8,45,46]. Thus, this range of temperatures was selected to capture this species' calcification response to the temperature variability occurring at present within a given year, as well as to the range of average annual seawater temperatures predicted for the next century. Coral specimens were reared in triplicate glass tanks at each of the three temperatures (nine tanks total). Mixed gas with an average *p*CO_2_ (s.d.) of 488 (88) µatm was bubbled with microporous ceramic airstones into the tanks. The *p*CO_2_ of the temperature treatments were slightly higher than present-day atmospheric value of 400 µatm due to slightly elevated *p*CO_2_ in the aquarium culture laboratory. Nevertheless, the pH range in the temperature experiment (7.9–8.0) was within the range observed for present-day reefs [47].

### (c) Tank conditions

Seawater within each tank was continuously filtered (757 l h^−1^) with a power filter. Circulation and turbulence of seawater was enhanced with a 400 l h^−1^ powerhead. Each tank was covered with a transparent 3-mm Plexiglas sheet and both the tank and filtration system were wrapped with cellophane to promote equilibration between the gas mixtures and the experimental seawaters and to minimize evaporative water loss. The tanks were illuminated for 12 h each day with compact fluorescent lights (ultra-actinic and white; 96 W, 10 000 K) and with standard white fluorescent lights (32 W, T8 6500 K), with a maximum photosynthetically active radiation (PAR) of *ca* 250 µmol photons m^−2^ s^−1^. The intensity and timing of the prescribed irradiance within the tanks was designed to replicate the light cycle of the corals' native habitat (see the electronic supplementary material for a detailed description of light conditions). PAR in the field and in the experimental tanks was measured using a LI-1400 datalogger affixed with a LI-192 underwater quantum sensor (LI-COR; Lincoln, Nebraska; see the electronic supplementary material, figures S1 and S2).

The nominal 28 and 32°C temperature treatments were maintained with 50-W heaters, while the 25°C treatment was maintained with a 1-hp aquarium chiller paired with a 50-W heater for stability. Seventy-five per cent seawater changes were performed weekly. Seawater pH and temperature returned to target values within 60 min of water changes. Each week, 250 ml seawater samples were obtained in ground-glass-stoppered borosilicate glass bottles for analysis of dissolved inorganic carbon and total alkalinity (TA). Seawater samples were obtained midway between weekly water changes in order to acquire average values for the water chemistry parameters in the treatment tanks. Small aliquots of deionized water were periodically added to the experimental tanks in order to replenish water lost through evaporation, thereby maintaining target salinity (35). Each coral specimen was hand-fed 20 mg of frozen *Artemia* sp. every other day using a 1-ml transfer pipette. Feeding trials conducted prior to the start of the experiment revealed that this amount of food was sufficient to adequately nourish the coral specimens.

### (d) Measurement and calculation of carbonate system parameters

Weekly seawater samples were analysed for DIC via coulometry (UIC 5400) and for TA via closed-cell potentiometric Gran titration calibrated with certified TA/DIC standards (see the electronic supplementary material for detailed methods). Temperature, salinity, and pH were determined via standard methods [48] approximately every other day. Additional carbonate system parameters (seawater *p*CO_2_, pH, carbonate ion concentration, bicarbonate ion concentration, aqueous CO_2_, and aragonite saturation state) were calculated with the program CO_2_SYS [49], using Roy *et al.* [50] values for *K*_1_ and *K*_2_ carbonic acid constants, the Mucci [51] value for the stoichiometric aragonite solubility product and an atmospheric pressure of 1.015 atm (see tables S1 and S2 and figures S3 and S4 of the electronic supplementary material for seawater chemistry data).

### (e) Quantification of calcification rates via buoyant weighing

*Siderastrea siderea* calcification rates were estimated using an empirically calibrated buoyant weight technique [14,52] (see the electronic supplementary material for empirical derivation of the buoyant weight–dry weight relationship for this species (figure S5)).

Calcification rates were estimated from the change in the coral specimen's dry weight normalized to its surface area and observational interval. Coral surface area was quantified from scaled top-view photographs of each coral specimen using the imaging software Image J.

### (f) Statistical analyses

Hierarchical mixed-effects models were employed to account for the combined repeated-measures/split-plot design to assess the overall effect of treatment on *S. siderea* calcification rates for the 95-day experiments and the impact of treatment duration on coral calcification response to warming and acidification (see the electronic supplementary material for details of statistical methods employed and tables S3 and S4 for description of observational intervals). All mixed models were estimated with the lme4 package [53] of *R* 3.0.2 [54].

Data are archived in the US National Science Foundation's Biological and Chemical Oceanography Database at (http://data.bco-dmo.org/jg/dir/test/OA_MarineCalcifiers/).

## 3. Results

### (a) Ocean acidification experiment

**Figure 1.**
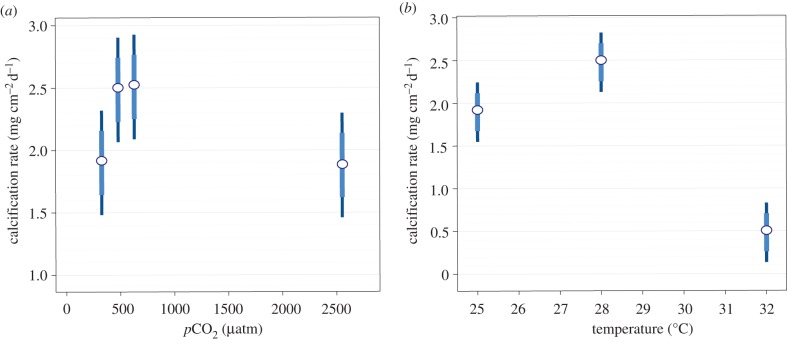
Parabolic calcification responses of the coral *S. siderea* to elevated *p*CO_2_ and temperature across the 95-day experiments. (*a*) Calcification rates for corals at mean *p*CO_2_ (s.d.) of 324 (89), 477 (83), 604 (107) and 2553 (506) µatm and at mean temperature (s.d.) of 28.10 (0.28)°C. (*b*) Calcification rates at mean temperatures (s.d.) of 25.01 (0.14), 28.16 (0.24) and 32.01 (0.17)°C and at mean *p*CO_2_ (s.d.) of 488 (88) µatm. Ninety-five per cent (thin bars) and 83.5% (thick bars) confidence intervals of the means are shown.

Calcification rates for the coral *S. siderea* exhibited a parabolic response to increasing atmospheric *p*CO_2_ (figure 1*a*). Over the entire 95-day experiment, calcification rates increased from the near-pre-industrial *p*CO_2_ value of 324 µatm to the near-present-day value of 477 µatm, remained relatively unchanged at the predicted end-of-century value of 604 µatm and returned to near-pre-industrial rates at six-times the modern *p*CO_2_ value of 2553 µatm (see the electronic supplementary material, table S5).

### (b) Temperature experiments

A parabolic calcification response pattern was also exhibited by the coral *S. siderea* in response to increasing seawater temperature (figure 1*b*). Over the entire 95-day experiment, calcification rates increased from the lower end of the corals' temperature range of 25°C to their average annual temperature of 28°C and then declined under a temperature of 32°C, near the upper end of their annual thermal range (see the electronic supplementary material, table S6).

### (c) Effect of exposure duration on coral calcification response to CO_2_-induced acidification and warming

**Figure 2.**
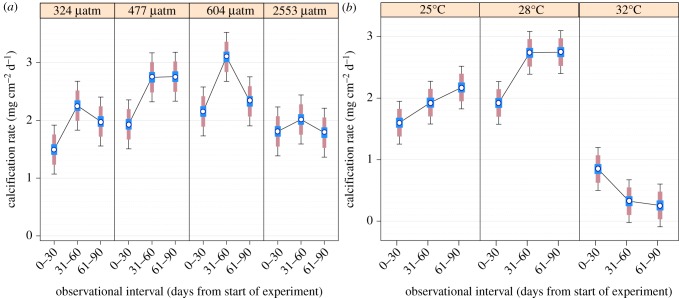
Effects of exposure duration on *S. siderea* coral calcification response to *p*CO_2_ and temperature. (*a*) Calcification rates at three monthly observational intervals for *S. siderea* corals reared at mean *p*CO_2_ (s.d.) of 324 (89), 477 (83), 604 (107) and 2553 (506) µatm and maintained at mean temperature (s.d.) of 28.10 (0.28)°C. (*b*) Calcification rates at three monthly observational intervals for *S. siderea* corals reared at temperatures (s.d.) of 25.01 (0.14), 28.16 (0.24) and 32.01 (0.17)°C and at mean *p*CO_2_ (s.d.) of 488 (88). Ninety-five per cent confidence intervals (black bars) show precision of estimated calcification rates. Eighty-three and one-half per cent confidence intervals (pink bars) are for across-panel (i.e. across treatment) comparison. Forty-two and one-half per cent confidence intervals (blue bars) are for within-panel (i.e. within treatment) comparison.

Differences in coral calcification rates were also assessed across three *ca* 30-day observational intervals (0–30, 31–60 and 61–90 days) using difference-adjusted confidence intervals [55,56] to assess the impact of duration of exposure to *p*CO_2_ (see figure 2*a* and electronic supplementary material, table S7) and temperature treatments (see figure 2*b* and electronic supplementary material, table S8) on *S. siderea* calcification rates.

#### (i) Ocean acidification experiment

Comparisons *within p*CO_2_ treatments (i.e. within-panel comparisons; figure 2*a*; confidence interval = blue bars) reveal that calcification rates for *S. siderea* corals reared at 324, 477, 604 and 2553 µatm increased significantly between the first observational interval (0–30 days) and the second observational interval (31–60 days), but declined (except for corals reared at 477 µatm, which remained constant) between the second observational interval and the third observation interval (61–90 days). Notably, calcification rates for the third observational interval were significantly greater than at the first observational interval for the two lowest *p*CO_2_ treatments, but not for the two highest *p*CO_2_ treatments.

Comparisons *between p*CO_2_ treatments (i.e. across-panel comparisons; figure 2*a*; confidence interval = pink bars) reveal that calcification response patterns to acidification are parabolic for each of the three observational intervals.

#### (ii) Temperature experiment

Comparisons *within* temperature treatments (i.e. within-panel comparisons; figure 2*b*; confidence interval = blue bars) reveal that calcification rates increased across the three observational intervals for corals reared at 25°C, increased across the first two observational intervals for corals reared at 28°C and decreased across the first two observational intervals for corals reared at 32°C. However, coral calcification rates were constant between the second and third observational intervals for corals reared at 28 and 32°C.

Comparisons *between* temperature treatments (i.e. across-panel comparisons; figure 2*b*; confidence interval = pink bars) reveal that calcification response patterns to warming are parabolic for each of the three observational intervals.

### (d) Effect of reef zone on coral calcification response to CO_2_-induced acidification and warming

Calcification rates of *S. siderea* corals were not significantly different across reef zones (i.e. forereef versus backreef versus near shore colonies) within any of the *p*CO_2_ or temperature treatments (see the electronic supplementary material, figures S7 and S8, and tables S9 and S10).

## 4. Discussion

### (a) Parabolic calcification response to acidification

Calcification rates within the coral *S. siderea* increased with moderate elevations in *p*CO_2_, but declined with extreme elevation, yielding a parabolic response to CO_2_-induced ocean acidification. Previous experimental studies, most of which did not use a pre-industrial *p*CO_2_ level, showed that corals exhibit either no response [16,25,30,40], a threshold-negative response [14] or a linear negative response to CO_2_-induced ocean acidification [15,16,20,25,30], although in a recent study the cold-water coral *Lophelia pertusa* exhibited slightly enhanced calcification under acidified conditions [57].

**Figure 3.**
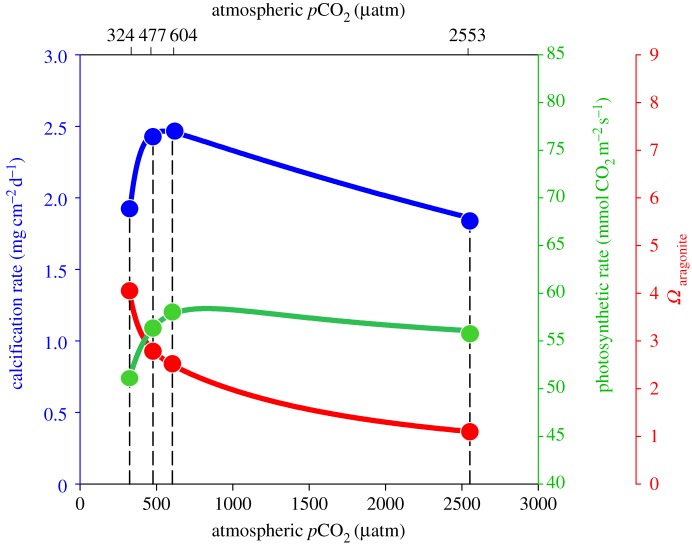
Conceptual diagram (constrained by study results) illustrating how photosynthesis (green curve; estimated from measured *F*_v_/*F*_m_ using empirical *F*_v_/*F*_m_–ETR_max_ relationship from Frade *et al.* [58] (see the electronic supplementary material, figure S6)) and aragonite saturation state (*Ω*_A_) (red curve; data from this study) interact to generate the corals' parabolic calcification response (blue curve; data from this study) to rising atmospheric *p*CO_2_.

There are two important factors involved in the process of coral calcification that are impacted by CO_2_-induced ocean acidification in potentially opposite ways: seawater saturation state with respect to the calcium carbonate mineral aragonite (*Ω*_A_) and photosynthesis (figure 3). Increasing *p*CO_2_ causes seawater pH to decline, which results in a reduction in carbonate ion concentration ([CO_3_^−2^]) and thus *Ω*_A_, which should impair calcification (red curve, figure 3). Conversely, increasing *p*CO_2_ causes the amount of CO_2_ dissolved in seawater, i.e. aqueous CO_2_ (CO_2_-aq), to increase, which should fertilize photosynthesis by the coral's algal symbionts, yielding more photosynthate and thus more energy for coral calcification [39] (green curve, figure 3). Recent studies on *Symbiodinium* phylotypes previously isolated from reef-building corals suggest that the diffusive uptake of CO_2_-aq from the external medium within at least one of four *Symbiodinium* phylotypes is at least partially dependent upon the concentration of CO_2_-aq [59,60]. Thus, CO_2_-induced ocean acidification may increase the concentration of CO_2_-aq available to this symbiont type, potentially elevating photosynthetic capacity of the coral holobiont that could confer supplemental energy for calcification.

A generalized model of the relationship between aragonite saturation state (red curve), rate of photosynthesis (green curve) and rate of coral calcification (blue curve)—each constrained by measurements from the present experiment (solid circles)—is rendered in figure 3. Aragonite saturation states and rates of calcification were measured directly, while rates of photosynthesis were estimated indirectly from pulse amplitude modulated fluorometry (see the electronic supplementary material (figure S6) for details of how photosynthetic rates were estimated). This analysis reveals that rates of symbiont photosynthesis (green curve in figure 3) increase with increasing *p*CO_2_ from 324 to 604 µatm, and then decline slightly between 604 and 2553 µatm. Thus, moderate elevations in *p*CO_2_ (324–604 µatm) appear to enhance photosynthesis of *Symbiodinium* within *S. siderea*, while extreme elevations cause symbiont photosynthesis to plateau or slightly decline [59], perhaps because CO_2_ is no longer limiting for photosynthesis at these elevated levels. The model (figure 3) suggests that calcification rates for *S. siderea* corals may increase (figure 1*a*) as *p*CO_2_ rises from 324 to 477 µatm because the challenge of calcifying under lower *Ω*_A_ is outweighed by the benefits of enhanced symbiont photosynthesis (e.g. increased energy and/or more favourable carbonate chemistry at the site of calcification) under moderately elevated *p*CO_2_ (324–477 µatm; figure 3). As *p*CO_2_ rises from 477 to 604 µatm, *Ω*_A_ continues to decrease while the benefits to calcification conferred by CO_2_-enhanced photosynthesis should continue to increase. It is therefore possible that the observed lack of change in coral calcification rate from 477 to 604 µatm (figure 1*a*) results from the benefit of enhanced photosynthesis being effectively neutralized, in terms of its impact on coral calcification rate, by the decline of *Ω*_A_ towards undersaturated conditions. Likewise, the increase in *p*CO_2_ from 604 µatm to the ultra-high value of 2553 µatm translates to an extreme decrease in *Ω*_A_—nearly to the point of undersaturation (*Ω*_A_ < 1)—which may outweigh the now relatively minor benefit of CO_2_-enhanced photosynthesis as the corals' symbionts transition away from strict CO_2_-limitation [30,61] (figure 3), resulting in the substantial decline in coral calcification rate observed across the 604–2553 µatm range (figure 1*a*).

The surprising ability of *S. siderea* corals to continue building new skeletal material under all experimental treatments, even at the nearly undersaturated (*Ω*_A_ < 1) level of 2553 µatm, may arise from the corals' capacity to manipulate the carbonate chemistry at their site of calcification [14,31,62–64]. Some calcifying organisms, by elevating pH of their calcifying fluid, facilitate the deprotonation of bicarbonate ions―whose concentrations are increased under conditions of elevated *p*CO_2_―resulting in elevated carbonate ion concentrations and *Ω*_A_ at the site of calcification. Indeed, *in situ* microelectrode measurements of pH within the calcifying medium of the tropical scleractinian coral *Galaxea fascicularis* reveal greater than one pH unit increase above that of ambient seawater [65]. Similar increases in pH have been measured within the calcifying fluid of the temperate scleractinian coral *Astrangia poculata* [15] and inferred for the tropical scleractinian corals *Stylophora pistillata* [66], *Porites* sp.[31], *Cladocora caespitosa* [67], *Desmophyllum dianthus* [68], *Favia fragum* [69] and various species of cold-water scleractinia [70]. A recent study also reveals spatial variations in the calcifying fluid pH of the coral *S. pistillata*, with polyp tissue exhibiting apparently greater control over calcifying fluid pH than coenosarc tissue [71].

Yet, despite the ability of *S. siderea* corals to continue building new skeletal material at *p*CO_2_ of 2553 µatm, the decline in calcification rate from 604 to 2553 µatm reveals there is a limit to the extent that they can manipulate carbonate chemistry at their site of calcification under conditions of elevated *p*CO_2_—beyond which coral calcification rates will decline.

### (b) Parabolic calcification response to warming

A parabolic response pattern was also exhibited by the *S. siderea* corals in response to increasing seawater temperature, with calcification increasing from 25 to 28°C, reaching a maximum at 28°C, and then decreasing from 28 to 32°C (figure 1*b*). This is consistent with a typical thermal performance curve, in which biological performance increases with rising temperature, reaches a maximum at an optimal temperature, and then declines as temperature continue to rise [72–74].

The parabolic shape of the thermal performance curve is usually attributed to a combination of thermodynamic effects of temperature on reaction rates and the destabilizing effects of temperature on a range of intermolecular interactions [75]. Specifically, the increase in coral calcification from 25 to 28°C may result from thermal acceleration of coral metabolism, including acceleration of zooxanthellate photosynthesis or increased rates of respiration by the coral animal, which would increase thermal energy (as described by the Arrhenius equation) and thus increase rates of chemical reactions involved in calcification [76]. The thermally driven increase in aragonite saturation state may also contribute to the increase in calcification rate observed between 25°C and 28°C. The waning phase of the thermal performance curve results from the destabilizing effects of temperature on a range of intermolecular interactions, ultimately leading to the destruction of the coral–dinoflagellate symbiosis—a process known as coral bleaching [13,77].

The parabolic shape of *S. siderea*'s calcification response to both warming and acidification suggests that parabolic responses to environmental stressors may be the norm and that linear responses arise when the range of the independent stress variable (e.g. temperature, *p*CO_2_) is too narrow to capture the full parabolic geometry of the response pattern. However, our observation that the calcification responses of *S. siderea* to both warming and acidification are parabolic does not necessarily mean that the corals' response to future combined warming and acidification will be parabolic.

Although target temperature and *p*CO_2_ levels were generally maintained throughout the 95-day experimental interval, there was moderate variability in TA and associated carbonate system parameters within both sets of experiments. These variations in TA were driven by progressive sequestration of carbonate ions through the coral calcification process. Although weekly water changes were performed, only 75% of the experimental seawater was exchanged in order to avoid shocking the corals. Thus, 25% of the TA drawdown was passed on to the next week's treatment, causing the weekly drawdown in TA to be semi-cumulative throughout the duration of the experiment. This resulted in two trends in TA among treatments: variability in weekly TA within treatments and variability in average TA among treatments (see the electronic supplementary material, tables S1 and S2, and figures S3 and S4).

These trends were most pronounced in the temperature experiment due to the relatively large difference in average calcification rates between the 32°C (TA = 2725 µM) and 28°C (TA = 1951 µM) treatments, which translated to proportional differences in TA (and associated carbonate system parameters) between the treatments. However, after controlling for the effect of temperature on pH, the elevated TA in the 32°C only imparts an approximately 0.1 unit effect on pH relative to pH of the 28°C treatment. Differences in calcification rates between the high-calcification-rate *p*CO_2_ treatments (i.e. 477, 604 µatm) and the low-calcification-rate *p*CO_2_ treatments (i.e. 324, 2553 µatm) yielded similar but more muted trends in TA for the *p*CO_2_ experiment.

Since elevated calcification was causing the decline in TA in both the temperature and *p*CO_2_ experiments (rather than depressed TA causing the decline in calcification), corals exhibiting the slowest calcification rates occupied treatments with the highest, most geochemically favourable TA. Therefore, it is reasonable to conclude that the observed differences in TA among treatments only dampened the fundamental calcification trends that were observed, rather than modifying their directions. Had the intermediate *p*CO_2_ and temperature treatments that supported the faster calcifying corals been fixed at the higher TAs that were maintained for the low and high *p*CO_2_ and temperature treatments, then the faster calcifying corals in the intermediate treatments would have experienced higher aragonite saturation states and thus presumably exhibited even higher calcification rates—thereby enhancing the parabolic shape of the calcification trends observed in both experiments.

Many studies on coral calcification [78,79] use such coral-induced drawdown of TA in a closed system to estimate coral calcifications rates (2 moles of TA = 1 mole of CaCO_3_ produced), an approach known as the ‘alkalinity anomaly technique’. Indeed, this approach is recommended as one of the ‘best practices' for quantifying calcification rates in ocean acidification research [80]. Nevertheless, the observed differences in coral-induced drawdown of TA and associated carbonate system parameters among treatments should be duly considered in the interpretation of these results.

### (c) Duration of exposure to CO_2_-induced acidification impacts coral calcification rate

The increase in calcification rates of *S. siderea* between the 0–30-day and the 31–60-day observational intervals suggests that the corals continued acclimating to their treatment conditions throughout these intervals (figure 2*a*), despite the prescribed acclimation period and gradual adjustment of temperature and *p*CO_2_ to the treatment levels. The difference in coral calcification rate between these two observational intervals suggests that a coral's response to an ocean acidification experiment is impacted by its duration of exposure, and may partly explain the wide range of calcification response patterns exhibited by identical or similar organisms in experiments that differ in their duration [14,16,22,23]. Despite these within-treatment differences in calcification rate across the three observational intervals, the corals exhibited comparably parabolic response patterns to acidification within each of the three observational intervals.

Although corals reared at 477 µatm maintained constant calcification after the second observational interval, calcification rates for corals reared at 324, 604 and 2553 µatm declined between the second and third observational intervals. Perhaps during shorter term exposure of these corals to elevated (604, 2553 µatm) or reduced (324 µatm) *p*CO_2_, the corals are able to maintain their calcifying medium at a suitable *Ω*_A_ via pH regulation of the calcifying medium [66], which requires energy. More prolonged exposure to *p*CO_2_ perturbation, however, may deplete the corals' lipid energy reserves, which would limit their ability to regulate *Ω*_A_ at the site of calcification, resulting in the reduced calcification rates evident in the third observational interval (61–90 days). *Siderastrea siderea* corals reared at the near-present-day *p*CO_2_ level of 477 µatm would have experienced the least change in energetic demands associated with regulating carbonate chemistry at their site of calcification, which is consistent with their calcification rates remaining constant between the second and third observational intervals. Although it is assumed that calcification consumes more energy under acidified conditions [23,37], a recent study [25] shows that lipid reserves of four coral species did not decline after approximately 30 days as *p*CO_2_ was elevated from 382 to 741 µatm. Thus, the findings of that study are not consistent with our assertion that *S. siderea* lipid reserves are progressively depleted when the corals are exposed to prolonged periods of acidification. These disparities may arise from interspecific differences in energetic demands of calcification or from differences in the duration of the corals' exposure to elevated *p*CO_2_.

### (d) Duration of exposure to warming impacts coral calcification rate

The increase in calcification rates across the three observational intervals for corals reared at 25°C suggests that they continued to acclimate to the low temperature conditions throughout the duration of the experiment. Conversely, the relative stabilization in calcification rates between the second and third observational intervals for corals reared at 28 and 32°C suggests that they had fully acclimated by the end of the second interval (figure 2*b*). Yet, despite these within-treatment differences in calcification rate across observational intervals, the corals' general calcification response patterns to warming were parabolic within each of the three observational intervals.

It is unlikely that the effects of exposure duration on the calcification response of *S. siderea* corals in this study simply arose from the corals' experimental conditions differing from their natural habitat as such effects should have been constant among treatments and thus impacted corals in all treatments in approximately the same manner. This was not borne out in the experiments, as exposure duration generally had less of an impact on corals in the control treatments than on corals in the high/low *p*CO_2_ and temperature treatments—suggesting that the variable effects of exposure duration were indeed linked to the experiments' independent variables (temperature and *p*CO_2_).

### (e) Near shore, backreef and forereef colonies exhibit equivalent responses to ocean acidification and warming

No statistically significant differences in calcification rates were observed among forereef, backreef and near shore colonies reared under replicate treatments in this study (see the electronic supplementary material, figures S7 and S8, and tables S9 and S10). However, it is possible that a longer experiment, across narrower ranges and finer increments of temperature, would reveal the differential responses among *S. siderea* corals from different reef zones that were evident in recently obtained cores of this species [8].

### (f) Ocean warming poses a more immediate threat than ocean acidification for the coral *Siderastrea siderea*

This experimental study shows that calcification rates of *S. siderea* corals exposed to IPCC projected end-of-century tropical seawater temperatures (32°C) declined nearly 80% relative to the control treatment (28°C), while calcification rates for corals reared at IPCC projected end-of-century *p*CO_2_ levels (604 µatm) were unchanged relative to the control treatment (477 µatm). Thus, given IPCC's projections for end-of-century climate and oceanic change [81], the results of this study suggest that ocean warming poses a more immediate threat than ocean acidification for the coral *S. siderea*. That said, interpretation of these isolated impacts of warming and acidification on coral calcification should be tempered by the understanding that these two stressors are occurring and will continue to occur in tandem.

## Supplementary Material

Electronic Supplementary Material for “The reef-building coral Siderastrea siderea exhibits parabolic responses to ocean acidification and warming”

## Data accessibility

Data are archived in the US National Science Foundation's Biological and Chemical Oceanography Database at (http://data.bco-dmo.org/jg/dir/test/OA_MarineCalcifiers/).
