# Supplementary material for: The reef-building coral Siderastrea siderea exhibits parabolic responses to ocean acidification and warming
Source: Proc Biol Sci. 2014 Dec 22;281(1797):20141856. doi: 10.1098/rspb.2014.1856 (PMC4240989; doi:10.1098/rspb.2014.1856)
Supplement: Electronic Supplementary Material for “The reef-building coral Siderastrea siderea exhibits parabolic responses to ocean acidification and warming” [file rspb20141856supp1.docx]

**Electronic Supplementary Material for “***The reef-building coral* Siderastrea siderea *exhibits parabolic responses to ocean acidification and warming”*

Karl D. Castillo, Justin B. Ries, John F. Bruno, and Isaac T. Westfield

**Description of coral collection sites**

Forereef and backreef colonies were collected from the seaward and landward sides, respectively, of the barrier reef’s crest, approximately 40 km west of the Belize coast in the Sapodilla Cayes Marine Reserve. Nearshore *S. siderea* colonies were obtained from fringing reefs within 10 km of the Belize coast in the Port Honduras Marine Reserve. Coral colonies were collected from sites at least 0.2 km apart in order to randomize microenvironmental and genetic effects. Required permits were obtained from the Belize Fisheries Department and all corals were collected pursuant to local, federal, and international regulations.

**Quantification of light conditions**

Photosynthetically active radiation (PAR) in the tanks was measured beneath the plexiglass cover using a *LI‑1400* datalogger affixed with a *LI‑192* underwater quantum sensor (*LI-COR*; Lincoln, Nebraska; see figure S2). PAR measurements in the field were performed across nearshore, backreef, and forereef collection sites during late May and early June of multiple years and ranged from 200 to 600 µmol photons m^-2^ s^-1^ at 3-5 meters depth. In addition, light intensity was recorded every 15 min using *HOBO Pendant* light dataloggers (*Onset Computer Corp.*; Bourne, Massachusetts; figure S1). The light dataloggers were cleaned regularly to minimize fouling. PAR was estimated from light intensity (lux) using the equation: 1µmol photons (400:700 nm) m^-2^ s^-1^ = 51.2 lux (sensu[^1^](#_ENREF_1)^,^[^2^](#_ENREF_2)). This light conversion was validated by mid-day measurements with the *LI-192* quantum sensor. Both the *HOBO* and *LI-192* light meters reveal that PAR values were often higher at offshore (forereef and backreef) sites compared to nearshore sites, with nearshore values generally falling within 100 to 400 µmol photons m^-2^ s^-1^ because of suspended materials within the water column. This trend was more pronounced during the rainy season, which extends from June to November off the coast of southern Belize, which can receive more than 4 m of rain annually. A conservative irradiation corresponding to the average 3-5 m deep nearshore values of 250 µmol photons m^-2^ s^-1^ was employed in order to minimize potential light stress.

**Measurement and calculation of carbonate system parameters**

Temperature within the experimental tanks was measured every other day with a NIST-calibrated partial-immersion organic‑filled glass thermometer (precision ±0.3%, accuracy ±0.4%). Salinity was measured every other day with a *YSI* 3200 conductivity meter with a *YSI* 3440 cell (K=10) calibrated with seawater standards (A. Dickson, Scripps Institute of Oceanography). Seawater pH was measured every other day with a *Thermo Scientific Orion 2 Star* benchtop pH meter with an *Orion* 9156BNWP pH probe, calibrated with 7.00 and 10.01 *Orion* NBS buffers traceable to NIST standard reference material (for slope of the calibration curve) and with seawater standards of known pH (A. Dickson, Scripps Institute of Oceanography; for y-intercept of the calibration curve). Seawater dissolved inorganic carbon (DIC) was measured via coulometry (*UIC 5400*) and total alkalinity (TA) was measured via closed-cell potentiometric Gran titration calibrated with certified Dickson TA/DIC standards (see Tables S1 and S2; Figures S3 and S4 for seawater chemistry data). Measurement of DIC and TA of the certified reference materials (CRMs) were consistently within 0.3% of certified values. Differences between the measured and certified TA and DIC values of the CRMs were used to correct measurements of experimental seawater solutions.

Seawater *p*CO_2_, pH, carbonate ion concentration ([CO_3_^2-^]), bicarbonate ion concentration ([HCO_3_^-^]), aqueous CO_2_, and aragonite saturation state (Ω_A_) were calculated from measured DIC, TA, temperature and salinity with the program CO_2_SYS [^3^](#_ENREF_3), using values for K_1_ and K_2_ carbonic acid constants [^4^](#_ENREF_4), the stoichiometric aragonite solubility product [^5^](#_ENREF_5), and an atmospheric pressure of 1.015 atm. Temporal variability in the prescribed carbonate system parameters throughout the duration of the experiments were generally consistent with that reported for natural reefs [^6^](#_ENREF_6).

**Empirical derivation of the buoyant weight-dry weight relationship**

*Siderastrea siderea* specimens were weighed at the beginning of the experiment and approximately every 30 days thereafter, with the final measurement obtained at 95 days. Each coral specimen was suspended by aluminum wire from a *Cole‑Parmer* bottom-loading scale (precision ±0.001; accuracy ±0.002) at 10 cm depth in a tank filled with experimental seawater maintained at 25 ºC and salinity of 33. A plastic-coated zinc mass standard was intermittently weighed to ensure consistency of the buoyant weight method.

The buoyant weight-dry weight relationship for the coral *S. siderea* was empirically derived by plotting the final dry weights (after removal of organic matter) against the final buoyant weights of 60 coral specimens randomly selected from the four *p*CO_2_ (324, 477, 604, 2553 µatm) and the three temperature (25, 28, 32 ºC) treatments employed in the experiments (Figure S5). The observation that specimens from all treatments are highly correlated (R^2^ = 0.9985, p <0.001) and fall on approximately the same line indicates that the density of the coral skeletons does not vary appreciably amongst treatments. Thus, a single linear equation can be used to convert buoyant weight to dry weight for the purposes of estimating net calcification rates:

Dry weight (mg) = 1.5567 * Buoyant weight (mg) + 1.1235.

**Additional explanations of the statistical model employed**

Hierarchical mixed‑effects models were employed to account for the combined repeated-measures/split-plot design. Each coral's measured dry weight (mg) was divided by its initial surface area (cm^2^) to yield a normalized weight (mg cm^-2^). In the *p*CO_2_ and temperature experiments, two different response variables were analyzed separately. Firstly, normalized dry weight (mg cm^-2^) was regressed against continuous time and treatment to assess the overall effect of treatment on *S. siderea* calcification rates for the 95-day experiments. This approach yields a coefficient of time in the regression model and the extent to which it varies by treatment. Normalized calcification rate (mg cm^-2^ d^-1^) was then obtained by extracting the regression coefficient of the continuous time variable in the model. Secondly, normalized calcification rates of each coral were assessed across three discrete observational intervals and regressed against treatment and time in order to assess the impact of treatment duration on coral calcification response to warming and acidification (see electronic supplementary material, tables S3 and S4). Difference-adjusted-confidence-intervals were used to reveal statistically significant differences amongst *p*CO_2_ and temperature treatments [^7^](#_ENREF_7)^,^[^8^](#_ENREF_8).

In the combined repeated-measures/split-plot design, tanks represent plots, and temperature and *p*CO_2_ represent whole-plot treatments, while reef zones of coral colonies represent split-plot treatments. Random effects at the colony level were employed to account for the grouping of corals by colony (genotype), and at the level of the individual coral specimen to account for repeated measures. Repeated measures are nested in coral units, which are nested in colony blocks and tank blocks, with tank and colony blocks crossed.

The full interaction model was fit using restricted maximum likelihood to obtain unbiased estimates of the variance components and parameter standard errors. The variance components of the model varied as the predictors of the model were fixed. AIC was used to determine which level-1 coefficients should be randomized at each level. The AIC-best random effects model was refit using the Satterthwaite approximation in *SAS/STAT* software (Version 9.3 of the *SAS* System for Windows [^9^](#_ENREF_9)) to evaluate statistical significance of individual model terms. Finally, Bayesian methods were employed to obtain Bayesian-credible and highest-probability density intervals for model parameters of interest. Markov chain Monte Carlo estimation of the Bayesian models was performed using *JAGS* 3.1.0 [^10^](#_ENREF_10).

**Estimation of photosynthetic rates.** One‑time measurements of the maximum photosynthetic efficiency of photosystem II (F_v_/F_m_; where F_v_ = F_m_ – F_o_; and F_v_, F_m_, and F_o_ are variable, maximum, and minimum fluorescence, respectively) were obtained on day 94 of the experiments with an underwater PAM fluorometer (saturation width 0.80 s of > 5000 µmol photon m^-2^ s^-1^ saturation light pulse; *Diving-PAM, Walz*, Germany) at 20:00 hours, two hours after daytime illumination ended to ensure that non‑photochemical quenching was suppressed and that the corals were adequately dark-adapted. Measured F_v_/F_m_ was converted to maximum photosynthetic rate (ETR_max_) using the approximately linear relationship between F_v_/F_m_ and ETR_max_ estimated from data reported in Frade et al. (2008) [^11^](#_ENREF_11) (ETR_max_ = −186.08*(F_v_/F_m_) + 172.64; R^2^ = 0.2553; linear regression without outlier in figure S6; table S11)—the only published dataset that the authors were able to identify that contained simultaneous measurements of F_v_/F_m_ and maximum photosynthetic rate (ETR_max_) for multiple species of tropical scleractinian corals across a range of light conditions.

**Supplementary Tables and Figures**

**Table S1.** Average calculated and measured parameters for *p*CO_2_ treatments: *p*CO_2_ of the mixed gases in equilibrium with the experimental seawaters (*p*CO_2 (gas-e)_); calculated pH (pH_c_); carbonate ion concentration ([CO_3_^2‒^]); bicarbonate ion concentration ([HCO_3_^‒^]); dissolved carbon dioxide ([CO_2_]_SW_); and aragonite saturation state (Ω_A_); temperature (T); salinity (Sal), measured pH (pH_m_), total alkalinity (TA), and dissolved inorganic carbon (DIC). “SE” is the standard error of the mean, “SD” is the standard deviation of the mean, and “*n*” is the sample size.

| *Calculated parameters* | | | | | |
| --- | --- | --- | --- | --- | --- |
| ***p*CO_2 (gas-e)_** | **(µatm)** | **324** | **477** | **604** | **2553** |
|  | SD | 89 | 83 | 107 | 506 |
|  | SE | 14 | 13 | 17 | 78 |
|  | Range | 125 - 487 | 356 - 615 | 418 - 812 | 1500 - 3364 |
|  | n | 42 | 42 | 42 | 42 |
| pH_c_ |  | 8.07 | 7.90 | 7.82 | 7.32 |
|  | SD | 0.12 | 0.13 | 0.13 | 0.14 |
|  | SE | 0.02 | 0.02 | 0.02 | 0.02 |
|  | Range | 7.81 - 8.23 | 7.62 - 8.05 | 7.48 - 7.96 | 6.99 - 7.49 |
|  | n | 42 | 42 | 42 | 42 |
| [CO_3_^2-^] | (µM) | 246 | 176 | 155 | 66 |
|  | SD | 94 | 66 | 60 | 26 |
|  | SE | 15 | 10 | 9 | 4 |
|  | Range | 85 - 372 | 55 - 270 | 38 - 232 | 18 - 100 |
|  | n | 42 | 42 | 42 | 42 |
| [HCO_3_^-^] | (µM) | 1480 | 1516 | 1605 | 2133 |
|  | SD | 378 | 320 | 405 | 408 |
|  | SE | 58 | 49 | 63 | 63 |
|  | Range | 825 - 1993 | 883 - 1969 | 896 - 2254 | 1318 - 2620 |
|  | n | 42 | 42 | 42 | 42 |
| [CO_2_]_SW_ | (µM) | 8.5 | 12.5 | 15.9 | 67.2 |
|  | SD | 2.4 | 2.2 | 2.8 | 13.2 |
|  | SE | 0.4 | 0.3 | 0.4 | 2.0 |
|  | Range | 3.2 - 12.9 | 9.4 - 16.0 | 11.0 - 21.3 | 39.6 - 88.6 |
|  | n | 42 | 42 | 42 | 42 |
| Ω_A_ |  | 4.0 | 2.8 | 2.5 | 1.1 |
|  | SD | 1.5 | 1.1 | 1.0 | 0.4 |
|  | SE | 0.2 | 0.2 | 0.1 | 0.1 |
|  | Range | 1.4 - 6.0 | 0.9 - 4.3 | 0.6 - 3.7 | 0.3 - 1.6 |
|  | n | 42 | 42 | 42 | 42 |
| *Measured parameters* | | | | | |
| T | (ºC) | 28.14 | 28.16 | 28.04 | 27.93 |
|  | SD | 0.27 | 0.24 | 0.28 | 0.19 |
|  | SE | 0.03 | 0.02 | 0.03 | 0.02 |
|  | Range | 27.70 - 28.80 | 27.80 - 28.90 | 27.50 - 28.90 | 27.60 - 28.50 |
|  | n | 108 | 108 | 108 | 108 |
| Sal |  | 35.04 | 35.01 | 35.11 | 35.04 |
|  | SD | 0.30 | 0.30 | 0.37 | 0.30 |
|  | SE | 0.03 | 0.03 | 0.03 | 0.03 |
|  | Range | 34.50 - 35.70 | 34.50 - 35.70 | 34.10 - 35.80 | 34.20 - 35.70 |
|  | n | 108 | 108 | 108 | 108 |
| pH_m_ |  | 8.05 | 7.93 | 7.83 | 7.35 |
|  | SD | 0.17 | 0.12 | 0.14 | 0.13 |
|  | SE | 0.01 | 0.01 | 0.01 | 0.01 |
|  | Range | 7.84 - 8.52 | 7.76 - 8.36 | 7.62 - 8.24 | 7.21 - 7.72 |
|  | n | 108 | 108 | 108 | 108 |
| TA | (µM) | 2086 | 1951 | 1986 | 2291 |
|  | SD | 554 | 458 | 532 | 458 |
|  | SE | 85 | 71 | 82 | 71 |
|  | Range | 1124 - 2702 | 1088 - 2601 | 1015 - 2760 | 1372 - 2786 |
|  | n | 42 | 42 | 42 | 42 |
| DIC | (µM) | 1735 | 1704 | 1776 | 2266 |
|  | SD | 459 | 381 | 463 | 428 |
|  | SE | 71 | 59 | 72 | 66 |
|  | Range | 937 - 2301 | 964 - 2249 | 956 - 2489 | 1412 - 2778 |
|  | n | 42 | 42 | 42 | 42 |

**Table S2.** Average measured and calculated parameters for temperature treatments: temperature (T); salinity (Sal); measured pH (pH_m_); total alkalinity (TA); dissolved inorganic carbon (DIC); *p*CO_2_ of the mixed gases in equilibrium with the experimental seawaters [*p*CO_2 (gas-e)_]; pH (pH_c_); carbonate ion concentration ([CO_3_^2‒^]); bicarbonate ion concentration ([HCO_3_^‒^]); dissolved carbon dioxide ([CO_2_]_SW_); and aragonite saturation state (Ω_A_). “SE” is the standard error of the mean, “SD” is the standard deviation of the mean, and “*n*” is the sample size.

| *Measured parameters* | | | | |
| --- | --- | --- | --- | --- |
| **T** | **(ºC)** | **25.01** | **28.16** | **32.01** |
|  | SD | 0.17 | 0.24 | 0.17 |
|  | SE | 0.01 | 0.02 | 0.02 |
|  | Range | 24.70 - 25.50 | 27.80 - 28.90 | 31.60 - 32.40 |
|  | n | 108 | 108 | 108 |
| Sal |  | 34.97 | 35.01 | 35.20 |
|  | SD | 0.30 | 0.29 | 0.32 |
|  | SE | 0.03 | 0.03 | 0.03 |
|  | Range | 34.40 - 35.90 | 34.50 - 35.70 | 34.40 - 36.20 |
|  | n | 108 | 108 | 108 |
| pH_m_ |  | 7.93 | 7.93 | 8.11 |
|  | SD | 0.10 | 0.13 | 0.07 |
|  | SE | 0.01 | 0.01 | 0.01 |
|  | Range | 7.86 - 8.27 | 7.76 - 8.36 | 8.09 - 8.42 |
|  | n | 108 | 108 | 108 |
| TA | (µM) | 2097 | 1951 | 2725 |
|  | SD | 448 | 458 | 446 |
|  | SE | 69 | 71 | 69 |
|  | Range | 1222 - 2728 | 1088 - 2601 | 1675 - 3217 |
|  | n | 42 | 42 | 42 |
| DIC | (µM) | 1859 | 1704 | 2284 |
|  | SD | 369 | 381 | 344 |
|  | SE | 57 | 59 | 53 |
|  | Range | 1145 - 2392 | 964 - 2249 | 1491 - 2683 |
|  | n | 42 | 42 | 42 |
| *Calculated parameters* | | | | |
| *p*CO_2 (gas-e)_ | (µatm) | 515 | 477 | 472 |
|  | SD | 92 | 83 | 86 |
|  | SE | 14 | 13 | 13 |
|  | Range | 365 - 694 | 356 - 615 | 319 - 694 |
|  | n | 42 | 42 | 42 |
| pH_c_ |  | 7.90 | 7.90 | 8.03 |
|  | SD | 0.13 | 0.12 | 0.11 |
|  | SE | 0.02 | 0.02 | 0.02 |
|  | Range | 7.59 - 8.04 | 7.62 - 8.05 | 7.70 - 8.15 |
|  | n | 42 | 42 | 42 |
| [CO_3_^2-^] | (µM) | 175 | 176 | 336 |
|  | SD | 67 | 66 | 93 |
|  | SE | 10 | 10 | 14 |
|  | Range | 52 - 263 | 55 - 270 | 113 - 463 |
|  | n | 42 | 42 | 42 |
| [HCO_3_^-^] | (µM) | 1669 | 1516 | 1936 |
|  | SD | 307 | 320 | 263 |
|  | SE | 47 | 49 | 41 |
|  | Range | 1072 - 2118 | 883 - 1969 | 1350 - 2313 |
|  | n | 42 | 42 | 42 |
| [CO_2_] _(SW)_ | (µM) | 14.6 | 12.5 | 11.3 |
|  | SD | 2.6 | 2.2 | 2.0 |
|  | SE | 0.4 | 0.3 | 0.3 |
|  | Range | 10.3 – 19.6 | 9.4 – 16.0 | 7.6 – 16.5 |
|  | n | 42 | 42 | 42 |
| Ω_A_ |  | 2.8 | 2.8 | 5.5 |
|  | SD | 1.1 | 1.1 | 1.5 |
|  | SE | 0.2 | 0.2 | 0.2 |
|  | Range | 0.8 - 4.2 | 0.9 - 4.3 | 1.9 - 7.6 |
|  | n | 42 | 42 | 42 |

**Table S3.** Observational intervals for *S. siderea* corals reared at the four *p*CO_2_ treatments levels. Buoyant weighing of the coral specimens was conducted over consecutive days, causing observational intervals to vary by a few days for corals in the different *p*CO_2_ treatments. To account for variability in the duration of these observational intervals, changes in corals’ surface-area-normalized dry weights (mg cm^-2^) were divided by the exact duration of their observational interval, thus yielding average daily calcification rates (mg cm^-2^ d^-1^).

| Cohort | *p*CO_2_ Treatment Tanks | Observation Interval | | | Number of  Corals | Percent of  Sample |
| --- | --- | --- | --- | --- | --- | --- |
|  |  | 1^st^ | 2^nd^ | 3^rd^ |  |  |
| 1 | 324 µatm (tanks 1-3)  604 µatm (tank 3) | [0,35]  35 days | [35, 60]  25 days | [60, 95]  35 days | 72 | 33.3 |
| 2 | 477 µatm (tanks 1-3) | [0,34]  34 days | [34, 60]  26 days | [60, 96]  34 days | 54 | 25.0 |
| 3 | 604 µatm (tanks 1 and 2) | [0,35]  35 days | [35, 60]  25 days | [60, 95]  35 days | 36 | 16.7 |
| 4 | 2553 µatm (tanks 1-3) | [0,33]  33 days | [33, 60]  27 days | [60,94]  34 days | 54 | 25.0 |

**Table S4.** Observational intervals for *S. siderea* corals reared at the three temperature levels. Buoyant weighing of the coral specimens was conducted over consecutive days, causing observational intervals to vary by a few days for corals in the different temperature treatments. To account for variability in the duration of these observational intervals, changes in corals’ surface-area-normalized dry weights (mg cm^-2^) were divided by the exact duration of their observational interval, thus yielding average daily calcification rates (mg cm^-2^ d^-1^).

| Cohort | Temperature Treatment Tanks | Observation Interval | | | Number of  Corals | Percent of  Sample |
| --- | --- | --- | --- | --- | --- | --- |
|  |  | 1^st^ | 2^nd^ | 3^rd^ |  |  |
| 1 | 25 ºC (tanks 1-3) | [0, 35]  35 days | [35, 60]  25 days | [60, 94]  34 days | 54 | 33.3 |
| 2 | 28 ºC (tanks 1-3) | [0, 34]  34 days | [34, 60]  26 days | [60, 96]  36 days | 54 | 33.3 |
| 3 | 32 ºC (tanks 1-3) | [0, 35]  35 days | [35, 60]  25 days | [60, 95]  35 days | 54 | 33.3 |

**Table S5.** Summary of mixed effects model to examine differences in calcification rates across *p*CO_2_ treatments with reefzone removed as a factor: covariance parameter estimates for random effects (a); Type 1 test of fixed effects using sequential variables-added-in-order-test (b); solutions for fixed effects (c); and 95% interval estimates of *p*CO_2_-time interaction effects in which 324 µatm is the reference group (d). Confidence intervals are obtained using Satterwaite correction and Bayesian model with uninformative priors using Markov chain Monte Carlo estimation. 95% credible intervals and highest posterior density intervals are shown.

**(a)**

| Covariance Parameter | Subject | Estimate |
| --- | --- | --- |
| (Intercept) | Coral ID | 0.04582 |
| Total Time | Coral ID | 0.08666 |
| (Intercept) | Colony | 0.01859 |
| Total Time | Colony | 0.1658 |
| Total Time | Tank | 0.07877 |
| Residual |  | 0.000159 |

**(b)**

| Parameter | Num DF | Den DF | *F-value* | *p-value* |
| --- | --- | --- | --- | --- |
| Total Time | 1 | 22.5 | 291.21 | <0.0001 |
| *p*CO_2_ | 3 | 198 | 2.34 | 0.0744 |
| Total Time: *p*CO_2_ | 6 | 8.01 | 4.33 | 0.0433 |

**(c)**

| Parameter | *p*CO_2_ | Estimate | SE | DF | *t-value* | *p-value* |
| --- | --- | --- | --- | --- | --- | --- |
| (Intercept) |  | 1.1867 | 0.04340 | 38.5 | 27.35 | <0.0001 |
| Total Time |  | 1.9004 | 0.1941 | 13.3 | 9.79 | <0.0001 |
| *p*CO_2_ | 2553 | 0.06985 | 0.04136 | 196 | 1.69 | 0.0928 |
| *p*CO_2_ | 604 | -0.06720 | 0.04153 | 197 | -1.62 | 0.1072 |
| *p*CO_2_ | 477 | -0.04390 | 0.04136 | 196 | -1.06 | 0.2898 |
| *p*CO_2_ | 324 | 0 | . | . | . | . |
| Total Time: *p*CO_2_ | 2553 | -0.02260 | 0.2387 | 8.01 | -0.09 | 0.9269 |
| Total Time: *p*CO_2_ | 604 | 0.6080 | 0.2387 | 8.02 | 2.55 | 0.0343 |
| Total Time: *p*CO_2_ | 477 | 0.5848 | 0.2386 | 8 | 2.45 | 0.0399 |
| Total Time: *p*CO_2_ | 324 | 0 | . | . | . | . |

**(d)**

| Interaction Effect | Bayesian Credible Intervals | Bayesian HPD Intervals |
| --- | --- | --- |
| 324 µatm | reference | reference |
| 477 µatm | (0.009, 1.173) | (0.008, 1.173) |
| 604 µatm | (0.015, 1.183) | (0.013, 1.175) |
| 2800 µatm | (–0.638, 0.556) | (–0.633, 0.561) |

Note: Intervals that do not include “zero” are significant.

**Table S6.** Summary of mixed effects model to examine differences in calcification rates across temperature treatments with reefzone removed as a factor: covariance parameter estimates for random effects (a); Type 1 test of fixed effects using sequential variables-added-in-order-test (b); solutions for fixed effects (c); and 95% interval estimates of *p*CO_2_-time interaction effects in which 25 ˚C is the reference group (d). Confidence intervals are obtained using Satterwaite correction and Bayesian model with uninformative priors using Markov chain Monte Carlo estimation. 95% credible intervals and highest posterior density intervals are shown.

**(a)**

| Covariance Parameter | Subject | Estimate |
| --- | --- | --- |
| (Intercept) | Coral ID | 0.04857 |
| Total Time | Coral ID | 0.07724 |
| (Intercept) | Colony | 0.009733 |
| Total Time | Colony | 0.07415 |
| (Intercept) | Tank | 0.000142 |
| Total Time | Tank | 0.05139 |
| Residual |  | 0.000144 |

**(b)**

| Parameter | Num DF | Den DF | *F-value* | *p-value* |
| --- | --- | --- | --- | --- |
| Total Time | 1 | 13.6 | 249.42 | <0.0001 |
| Temperature | 2 | 6.45 | 0.66 | 0.5469 |
| Total Time: Temperature | 2 | 6.01 | 55.01 | 0.0001 |

**(c)**

| Parameter | Temperature | | Estimate | SE | DF | *t-value* | *p-value* |
| --- | --- | --- | --- | --- | --- | --- | --- |
| (Intercept) | |  | 1.2141 | 0.03879 | 11.8 | 31.30 | <0.0001 |
| Total Time | |  | 1.8958 | 0.1526 | 8.71 | 12.42 | <0.0001 |
| Temperature | | 32 | 0.06170 | 0.04372 | 5.96 | 1.41 | 0.2082 |
| Temperature | | 28 | -0.07456 | 0.04366 | 5.93 | -1.71 | 0.1392 |
| Temperature | | 25 | 0 | . | . | . | . |
| Total Time: Temp | | 32 | -1.4150 | 0.1956 | 6.02 | -7.23 | 0.0003 |
| Total Time: Temp | | 28 | 0.5795 | 0.1956 | 6.01 | 2.96 | 0.0251 |
| Total Time: Temp | | 25 | 0 | . | . | . | . |

**(d)**

| Interaction Effect | Bayesian Credible Intervals | Bayesian HPD Intervals |
| --- | --- | --- |
| 25˚C | reference | reference |
| 28˚C | (0.037, 1.115) | (0.015, 1.093) |
| 32˚C | (–1.948, –0.881) | (–1.949, –0.881) |

Note: Intervals that do not include “zero” are significant.

**Table S7.** Summary of mixed-effects model to examine differences in calcification rates across temperature treatments with reefzone removed as a factor: covariance parameter estimates for random effects (a); Type 1 test of fixed effects using sequential variables-added-in-order-test (b); and solutions for fixed-effects (c).

**(a)**

| Covariance Parameter | Subject | Estimate |
| --- | --- | --- |
| (Intercept) | Coral ID | 0.05091 |
| (Intercept) | Colony | 0.16370 |
| (Intercept) | Tank | 0.07557 |
| Residual |  | 0.19270 |

**(b)**

| Parameter | Num DF | Den DF | *F-value* | *p-value* |
| --- | --- | --- | --- | --- |
| Time | 2 | 424 | 127.48 | <0.0001 |
| *p*CO_2_ | 3 | 8.01 | 4.50 | 0.0394 |
| Time: *p*CO_2_ | 6 | 424 | 16.08 | <0.0001 |

**(c)**

| Parameter | *p*CO_2_ | Time | Estimate | SE | DF | *t-value* | *p-value* |
| --- | --- | --- | --- | --- | --- | --- | --- |
| (Intercept) |  |  | 1.4781 | 0.1970 | 15.2 | 7.50 | <0.0001 |
| Time |  | 90 | 0.4803 | 0.08448 | 424 | 5.69 | <0.0001 |
| Time |  | 60 | 0.7510 | 0.08448 | 424 | 8.89 | <0.0001 |
| Time |  | 30 | 0 | . | . | . | . |
| *p*CO_2_ | 2553 |  | 0.3115 | 0.2438 | 9.46 | 1.28 | 0.2319 |
| *p*CO_2_ | 604 |  | 0.6523 | 0.2439 | 9.47 | 2.67 | 0.0244 |
| *p*CO_2_ | 477 |  | 0.4321 | 0.2438 | 9.46 | 1.77 | 0.1085 |
| *p*CO_2_ | 324 |  | 0 | . | . | . | . |
| Time: *p*CO_2_ | 2553 | 90 | -0.5025 | 0.1195 | 424 | -4.21 | <0.0001 |
| Time: *p*CO_2_ | 2553 | 60 | -0.5476 | 0.1195 | 424 | -4.58 | <0.0001 |
| Time: *p*CO_2_ | 2553 | 30 | 0 | . | . | . | . |
| Time: *p*CO_2_ | 604 | 90 | -0.3066 | 0.1195 | 424 | -2.57 | 0.0106 |
| Time: *p*CO_2_ | 604 | 60 | 0.1844 | 0.1195 | 424 | 1.54 | 0.1234 |
| Time: *p*CO_2_ | 604 | 30 | 0 | . | . | . | . |
| Time: *p*CO_2_ | 477 | 90 | 0.3364 | 0.1195 | 424 | 2.82 | 0.0051 |
| Time: *p*CO_2_ | 477 | 60 | 0.05461 | 0.1195 | 424 | 0.46 | 0.6478 |
| Time: *p*CO_2_ | 477 | 30 | 0 | . | . | . | . |
| Time: *p*CO_2_ | 324 | 90 | 0 | . | . | . | . |
| Time: *p*CO_2_ | 324 | 60 | 0 | . | . | . | . |
| Time: *p*CO_2_ | 324 | 30 | 0 | . | . | . | . |

**Table S8.** Summary of mixed-effects model to examine differences in calcification rates across temperature treatments with reefzone removed as a factor: covariance parameter estimates for random effects (a); Type 1 test of fixed-effects using sequential variables-added-in-order-test (b); and solutions for fixed effects (c).

**a.**

| Covariance Parameter | Subject | Estimate |
| --- | --- | --- |
| (Intercept) | Coral ID | 0.05650 |
| (Intercept) | Colony | 0.07322 |
| (Intercept) | Tank | 0.04854 |
| Residual |  | 0.1381 |

**b.**

| Parameter | Num DF | Den DF | *F-value* | *p-value* |
| --- | --- | --- | --- | --- |
| Time | 2 | 318 | 22.56 | <0.0001 |
| Temperature | 2 | 6.01 | 56.93 | 0.0001 |
| Time: Temperature | 4 | 318 | 67.35 | <0.0001 |

**c.**

| Parameter | Temperature | | Time | Estimate | SE | DF | *t-value* | *p-value* |
| --- | --- | --- | --- | --- | --- | --- | --- | --- |
| (Intercept) | |  |  | 1.9005 | 0.1546 | 10.2 | 12.30 | <0.0001 |
| Time | |  | 90 | 0.8167 | 0.07153 | 318 | 11.42 | <0.0001 |
| Time | |  | 60 | 0.8056 | 0.07153 | 318 | 11.26 | <0.0001 |
| Time | |  | 30 | 0 | . | . | . | . |
| Temperature | | 32 |  | -1.0620 | 0.1990 | 7.19 | -5.34 | 0.0010 |
| Temperature | | 25 |  | -0.3174 | 0.1990 | 7.19 | -1.60 | 0.1536 |
| Temperature | | 28 |  | 0 | . | . | . | . |
| Time: Temperature | | 32 | 90 | -1.4030 | 0.1012 | 318 | -13.87 | <0.0001 |
| Time: Temperature | | 32 | 60 | -1.3231 | 0.1012 | 318 | -13.08 | <0.0001 |
| Time: Temperature | | 32 | 30 | 0 | . | . | . | . |
| Time: Temperature | | 25 | 90 | -0.2520 | 0.1012 | 318 | -2.49 | 0.0133 |
| Time: Temperature | | 25 | 60 | -0.4835 | 0.1012 | 318 | -4.78 | <0.0001 |
| Time: Temperature | | 25 | 30 | 0 | . | . | . | . |
| Time: Temperature | | 28 | 90 | 0 | . | . | . | . |
| Time: Temperature | | 28 | 60 | 0 | . | . | . | . |
| Time: Temperature | | 28 | 30 | 0 | . | . | . | . |

**Table S9.** Summary of mixed-effects model to examine differences in calcification rates across reefzones within *p*CO_2_ treatments: covariance parameter estimates for random effects (a) and Type 1 test of fixed-effects using sequential-variables-added-in-order test (b).

**(a)**

| Covariance Parameter | Subject | Estimate |
| --- | --- | --- |
| (Intercept) | Coral ID | 0.04587 |
| Total Time | Coral ID | 0.08786 |
| (Intercept) | Colony | 0.02061 |
| Total Time | Colony | 0.19000 |
| Total Time | Tank | 0.07807 |
| Residual |  | 0.00015 |

**(b)**

| Parameter | Num DF | Den DF | *F-value* | *p-value* |
| --- | --- | --- | --- | --- |
| Total Time | 1 | 21.6 | 269.54 | <0.0001 |
| *p*CO_2_ | 3 | 195 | 2.24 | 0.0846 |
| Time: *p*CO_2_ | 3 | 8.03 | 4.35 | 0.0425 |
| Reefzone | 2 | 15.8 | 0.32 | 0.7332 |
| Total Time: Reefzone | 2 | 15.1 | 0.01 | 0.9873 |
| *p*CO_2_: Reefzone | 6 | 191 | 0.93 | 0.4724 |
| Total Time: *p*CO_2_: Reefzone | 6 | 182 | 0.69 | 0.6585 |

**Table S10.** Summary of mixed-effects model to examine differences in calcification rates across reefzones within temperature treatments: covariance parameter estimates for random effects (a) and Type 1 test of fixed-effects using sequential-variables-added-in-order-test (b).

**(a)**

| Covariance Parameter | Subject | Estimate |
| --- | --- | --- |
| (Intercept) | Coral ID | 0.04974 |
| Total Time | Coral ID | 0.07665 |
| (Intercept) | Colony | 0.01168 |
| Total Time | Colony | 0.08155 |
| (Intercept) | Tank | 0.00005 |
| Total Time | Tank | 0.05215 |
| Residual |  | 0.00014 |

**(b)**

| Parameter | Num DF | | Den DF | | *F-value* | | *p-value* | |
| --- | --- | --- | --- | --- | --- | --- | --- | --- |
| Total Time | | 1 | | 13.7 | | 238.40 | | <0.0001 |
| Temperature | | 2 | | 6.42 | | 0.66 | | 0.5490 |
| Total Time: Temperature | | 2 | | 6.02 | | 54.41 | | 0.0001 |
| Reefzone | | 2 | | 15.5 | | 0.01 | | 0.9921 |
| Total Time: Reefzone | | 2 | | 14.6 | | 0.25 | | 0.7840 |
| Temperature: Reefzone | | 4 | | 135 | | 0.25 | | 0.9102 |
| Total Time: Temperature: Reefzone | | 4 | | 132 | | 1.19 | | 0.3196 |

**Table S11.** Average measured F_v_/F_m_ obtained on day 94 of the experiment and maximum photosynthetic rate (ETR_max_) estimated using the approximately linear relationship between F_v_/F_m_ and ETR_max_ (ETR_max_ = −186.08*(F_v_/F_m_) + 172.64; R^2^ = 0.2553) from data reported in Frade et al. (2008; see Figure S6).

| *p*CO_2_ (µatm) | Mean F_v_/F_m_(dusk) | Maximum Photosynthetic Rate (ETR_max_; µmol CO_2_ m^-2^ s^-1^) |
| --- | --- | --- |
| 324 | 0.6527 | 51.1780 |
| 477 | 0.6249 | 56.3607 |
| 604 | 0.6169 | 57.8320 |
| 2553 | 0.6264 | 56.0746 |
|  |  |  |

**Figure S1.** Photosynthetic active radiation (PAR) measurements (December 2009 to September 2010) for offshore and nearshore sites where *Siderastrea siderea* corals were collected for the *p*CO_2_ and temperature experiments.

**Figure S2.** Twelve-hour light cycle for *Siderastrea siderea* corals maintained under the four *p*CO_2_ (324, 477, 604, 2553 µatm) and the three temperature (25, 28, 32 ºC) treatments over the 95-day experimental interval.

**
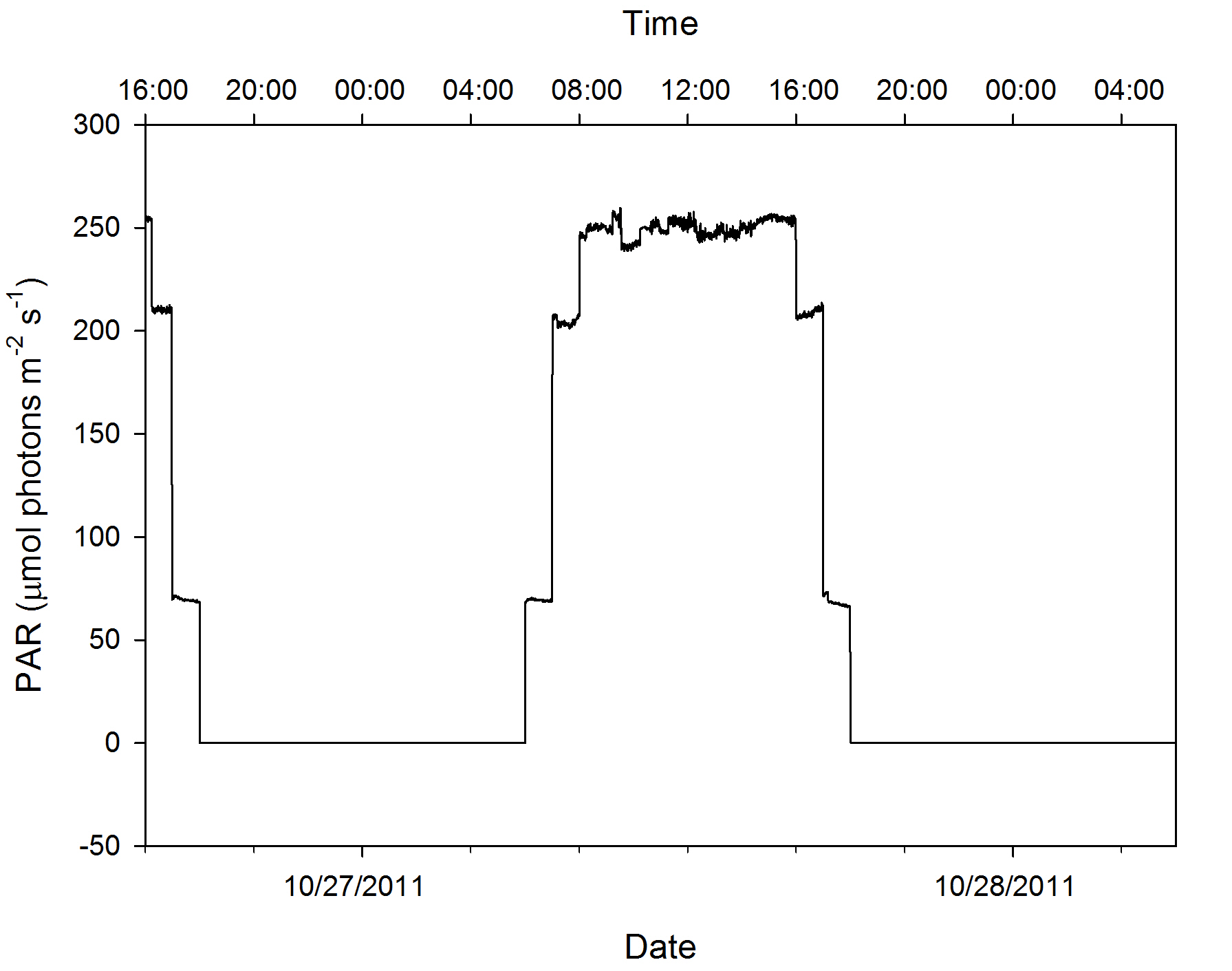
**

**Figure S3.** Calculated and measured parameters for *p*CO_2_ treatments over the 95-day experimental interval: calculated *p*CO_2_ of the mixed gases in equilibrium with the experimental seawaters (a); calculated pH (b); calculated carbonate ion concentration (c); calculated bicarbonate ion concentration (d); calculated dissolved carbon dioxide (e); calculated aragonite saturation state (f); measured temperature (g); measured salinity (h); measured pH (i); measured total alkalinity (j); and measured dissolved inorganic carbon (k).


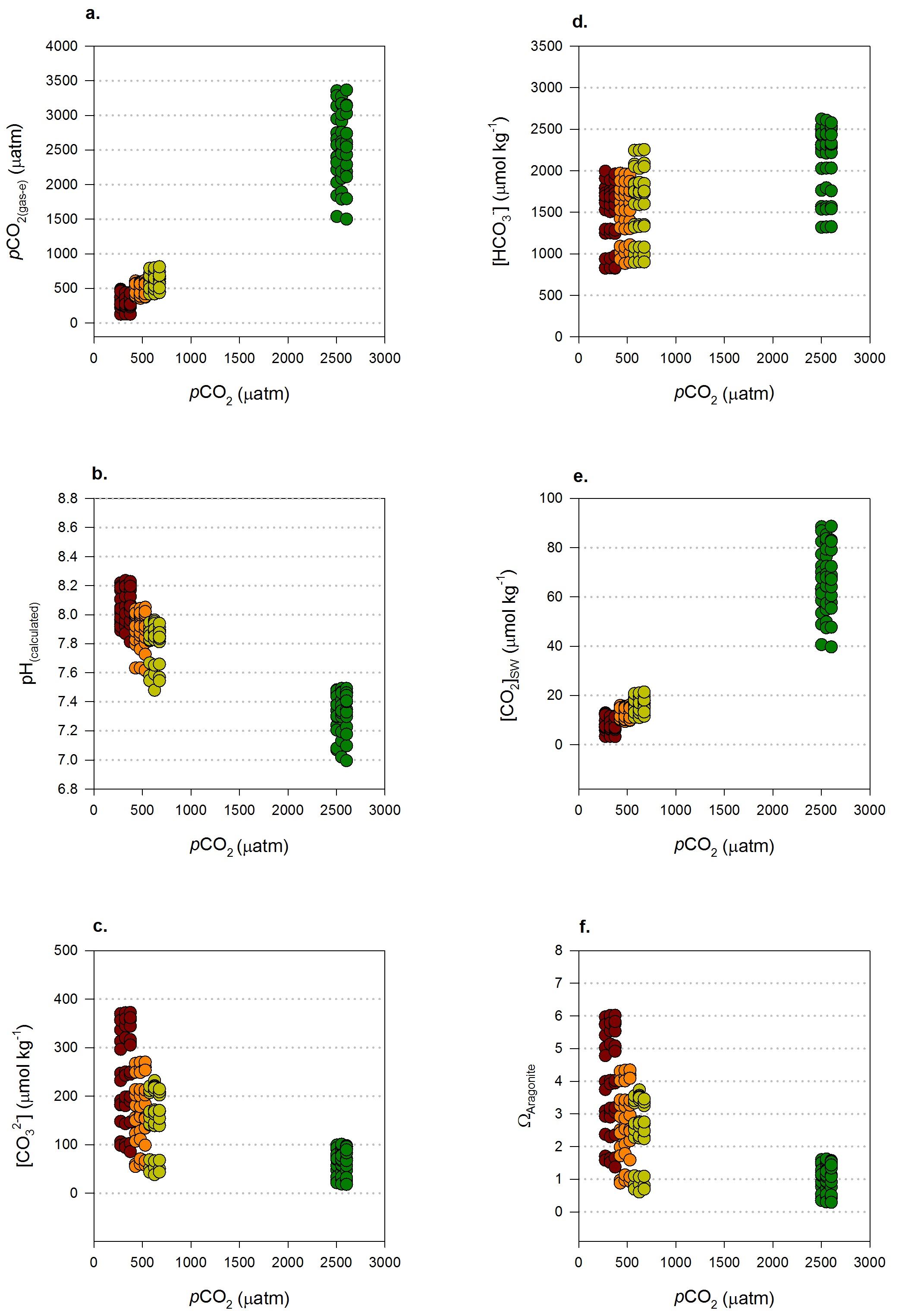


**
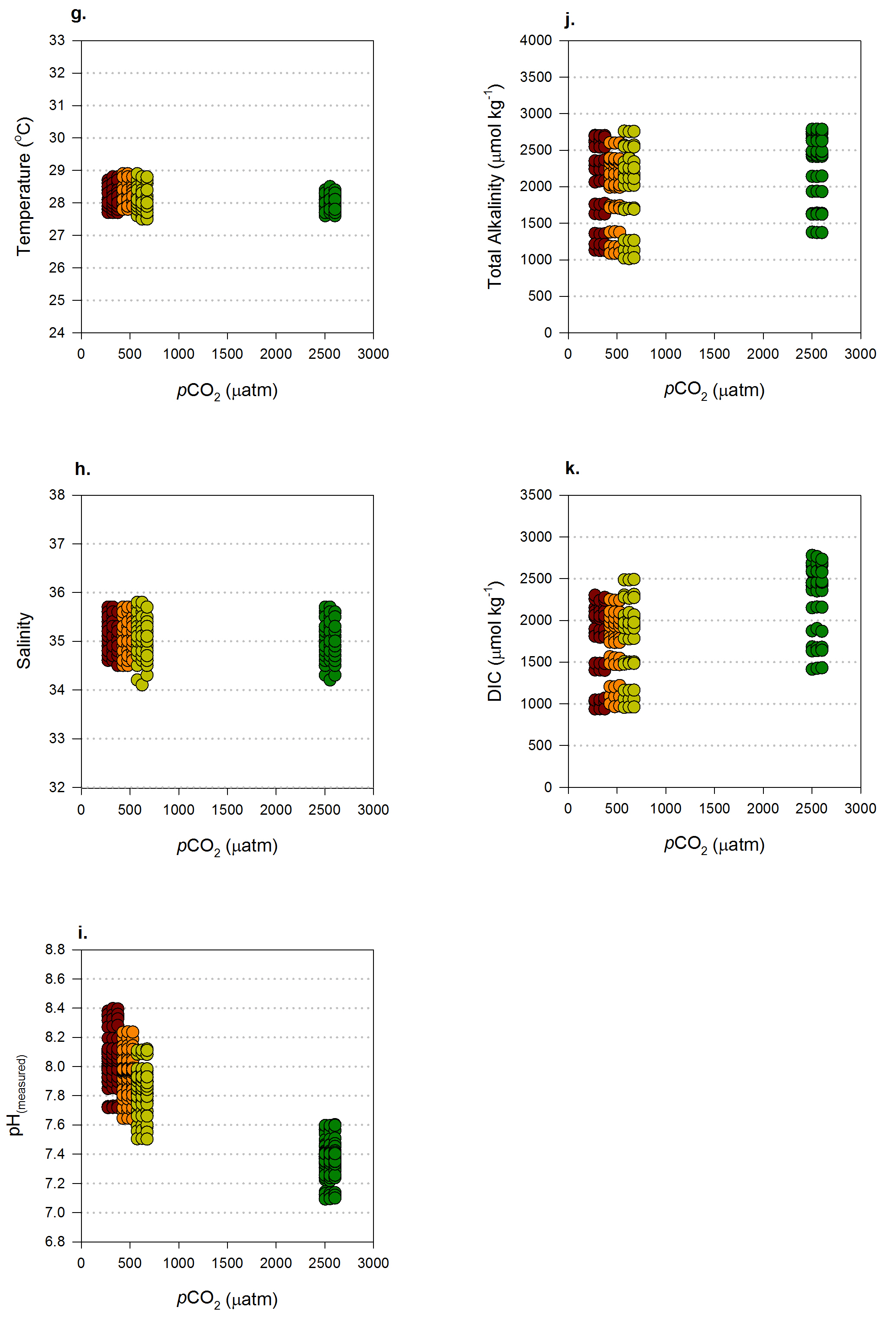
Figure S4.** Measured and calculated parameters for temperature treatments over the 95-day experimental interval: measured temperature (a); measured salinity (b); measured pH (c); measured total alkalinity (d); measured dissolved inorganic carbon (e); calculated *p*CO_2_ of the mixed gases in equilibrium with the experimental seawaters (f); calculated pH (g); calculated carbonate ion concentration (g); calculated bicarbonate ion concentration (i); calculated dissolved carbon dioxide (j); and calculated aragonite saturation state (k).

**
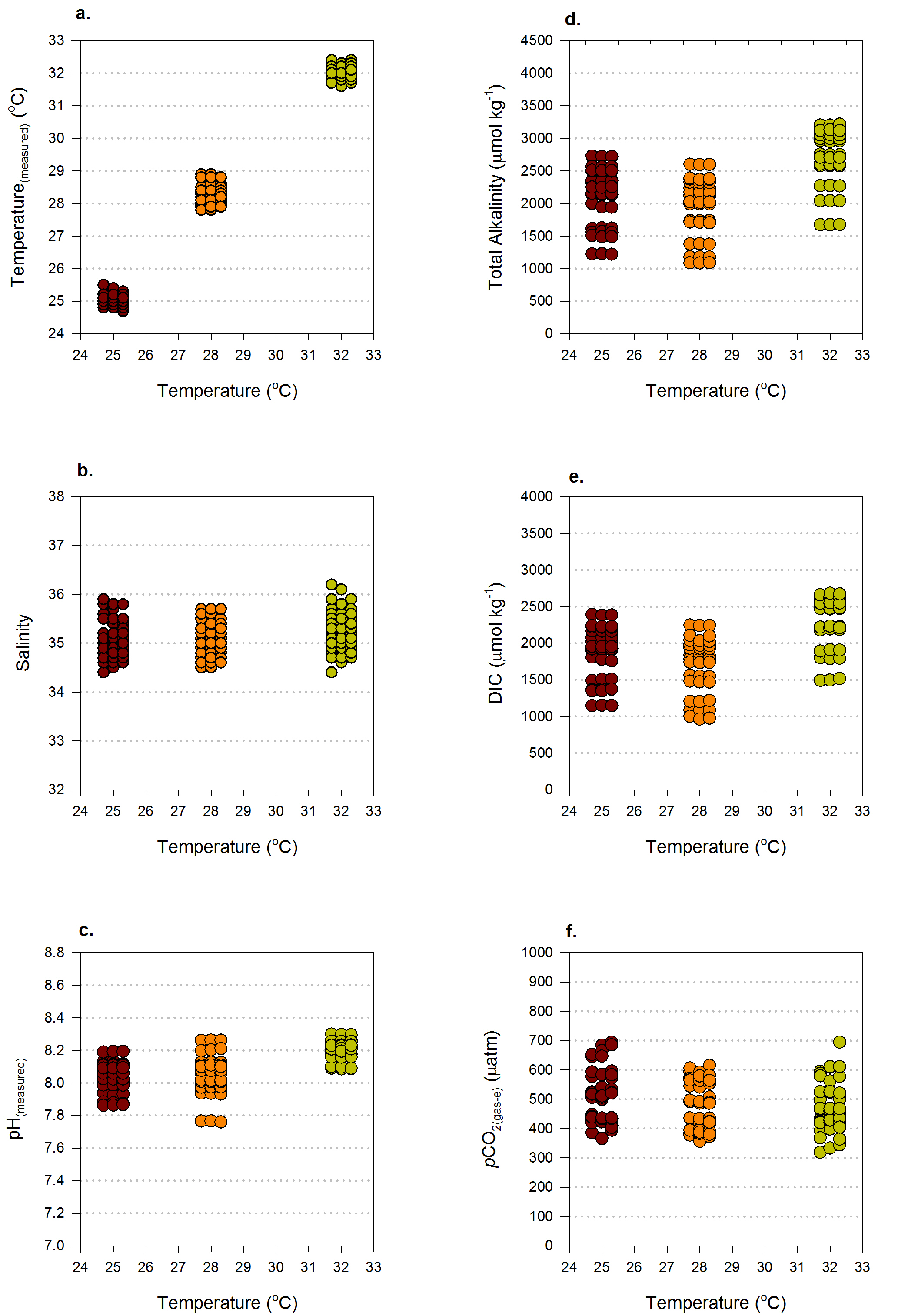
**

**
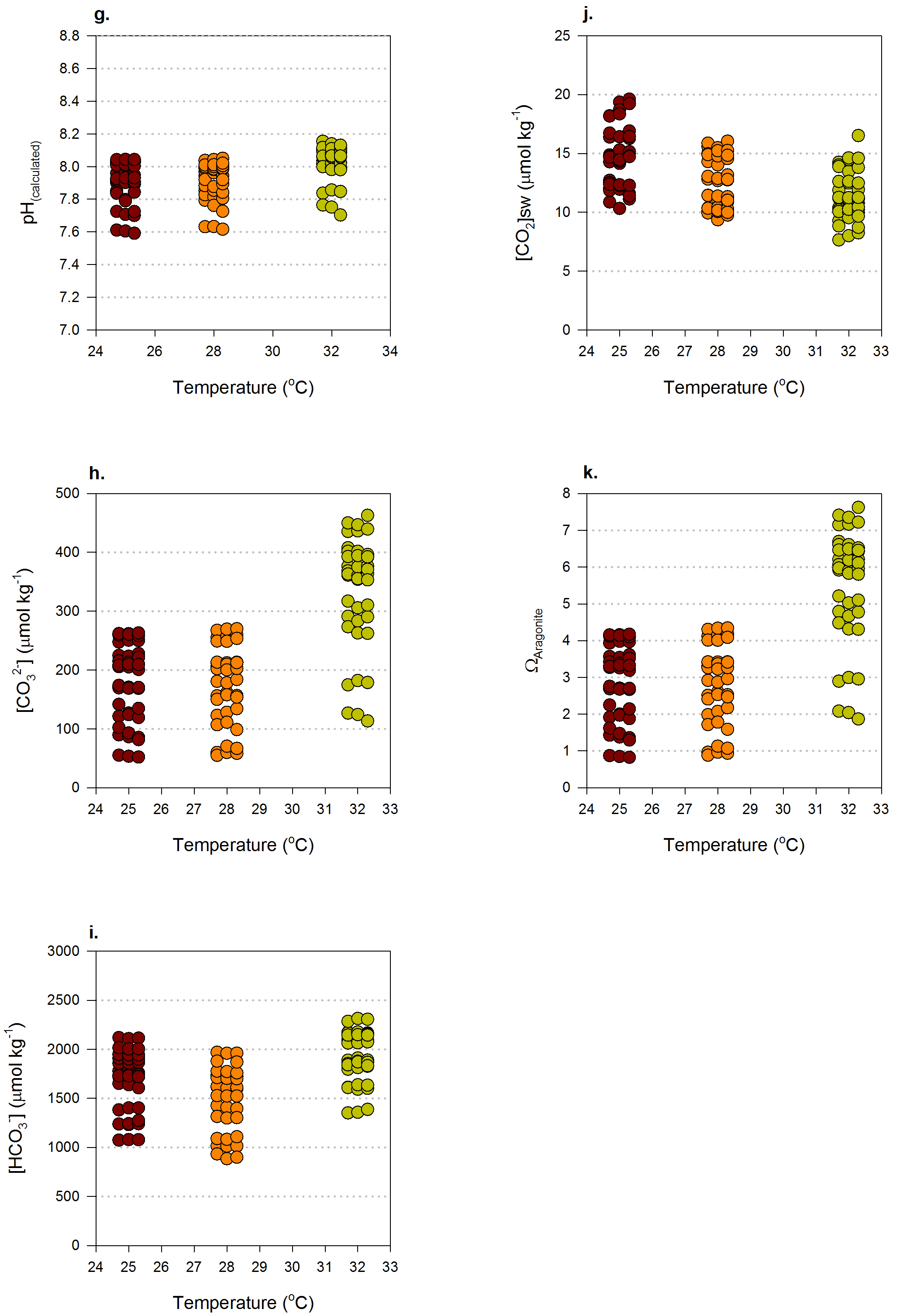
**


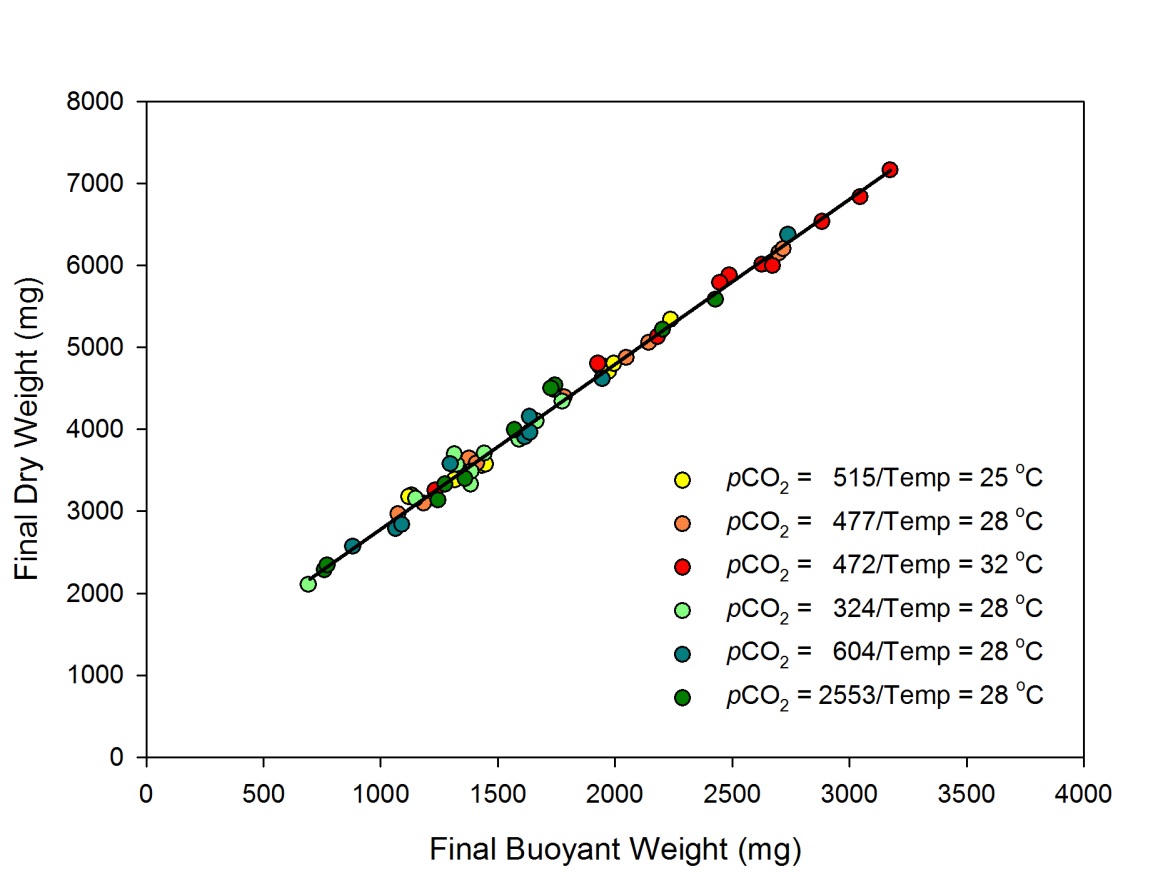
**Figure S5.** Final buoyant weight vs. final dry weight for 60 randomly selected *S. siderea* coral specimens that were reared in the various *p*CO_2_ and temperature treatments. The ‘*p*CO_2_ = 477/Temp = 28 ºC’ treatment was used in both the *p*CO_2_ and temperature experiments.

**Figure S6.** Relationship between maximum photosynthetic rate (ETR_max_) and maximum photochemical efficiency of photosystem II (F_v_/F_m_) at dusk for several species of corals (data from Frade et al., 2008). Maximum photosynthetic rate (ETR_max_) was estimated for corals in the present study (see Table S11; Figure 3) from average measured F_v_/F_m_ using the approximately linear relationship (excluding the outlier) between Frade et al.’s (2008) F_v_/F_m_ and ETR_max_ data (black line; ETR_max_ = −186.08*(F_v_/F_m_) + 172.64).

**
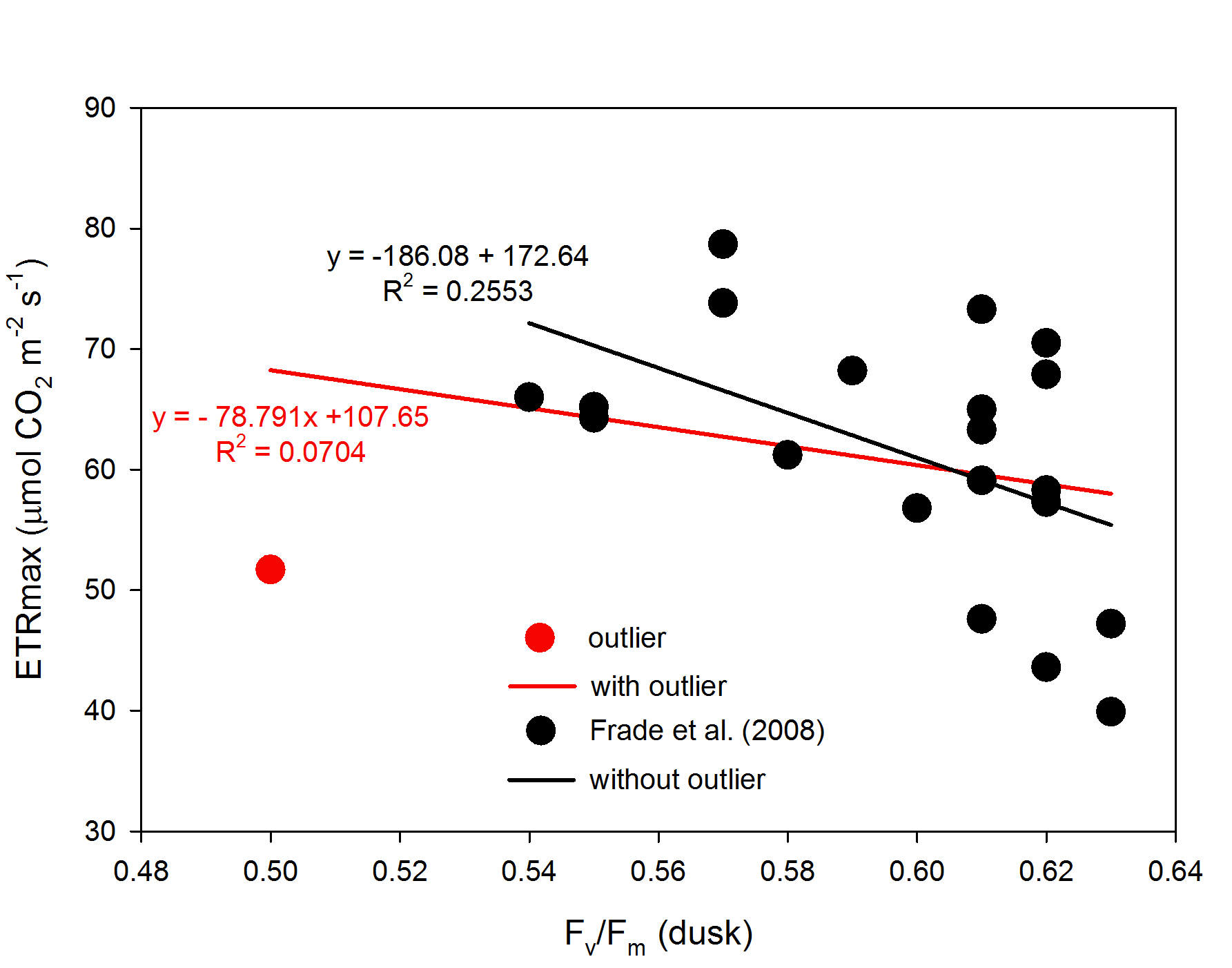
**

**Figure S7.** Average calcification rates for *S. siderea* coral specimens from the three reefzones (forereef, backreef, nearshore) reared at the four *p*CO_2_ levels. The 95% confidence intervals (thin bars) and 73% confidence intervals (thick bars) can be used for making pairwise comparisons amongst corals from the different reefzones. Neither set of confidence intervals reveals a statistically significant difference in calcification rates amongst corals from the different reefzones reared within the same *p*CO_2_ treatment.

**Figure S8.** Average calcification rates for *S. siderea* coral specimens from the three reefzones (forereef, backreef, nearshore) reared at the three temperature levels. The 95% confidence intervals (thin bars) and 73% confidence intervals (thick bars) can be used for making pairwise comparisons amongst reefzones. Neither set of confidence intervals reveals a statistically significant difference in calcification rates amongst corals from the different reefzones reared within the same temperature treatment.****

Supplementary References

1 Valiela, I. *Marine Ecological Processes*. (Springer, 1984).

2 Williams, G. J. *et al.* Ocean warming and acidification have complex interactive effects on the dynamics of a marine fungal disease. *Proceedings of the Royal Society B: Biological Sciences* 281, doi:10.1098/rspb.2013.3069 (2014).

3 Lewis, E. & Wallace, D. CO_2_SYS: Program developed for CO_2_ system calculations, ORNL/CDIAC-105. (1998).

4 Roy, R. *et al.* The dissociation constants of carbonic acid in seawater at salinities 5 to 45 and temperatures 0 to 45 ^o^C. *Marine Chemistry* 44, 249-267, doi:10.1016/0304-4203(93)90207-5 (1993).

5 Mucci, A. The solubility of calcite and aragonite in seawater at various salinities, temperatures, and one atmosphere total pressure. *American Journal Science* 283, 780-799, doi:10.2475/ajs.283.7.780 (1983).

6 Hofmann, G. E. *et al.* High-Frequency Dynamics of Ocean pH: A Multi-Ecosystem Comparison. *PLoS ONE* 6, e28983, doi:10.1371/journal.pone.0028983 (2011).

7 Baguley, T. *Serious stats: A guide to advanced statistics for the behavioral sciences*. (Basingstoke: Palgrave, 2012).

8 Baguley, T. Calculating and graphing within-subject confidence intervals for ANOVA. *Behaviour Research Methods* 44, 158-175, doi:10.3758/s13428-011-0123-7 (2012).

9 SAS/STAT 13.1 User's Guide (SAS Institute Inc.), Cary, NC, 2013).

10 Plummer, M. in *Proceedings of the 3rd International Workshop on Distributed Statistical Computing, .* 20-22.

11 Frade, P., Bongaerts, P., Winkelhagen, A., Tonk, L. & Bak, R. In situ photobiology of corals over large depth ranges: A multivariate analysis on the roles of environment, host, and algal symbiont. *Limnology and Oceanography* 53, 2711-2723, doi:10.4319/lo.2008.53.6.2711 (2008).
